# The Diversity of the Clinical Phenotypes in Patients With Fibrodysplasia Ossificans Progressiva

**DOI:** 10.14740/jocmr2465w

**Published:** 2016-01-26

**Authors:** Ali Al Kaissi, Vladimir Kenis, Maher Ben Ghachem, Jochen Hofstaetter, Franz Grill, Rudolf Ganger, Susanne Gerit Kircher

**Affiliations:** a Ludwig Boltzmann Institute of Osteology at the Hanusch Hospital of WGKK and AUVA Trauma Centre Meidling, First Medical Department, Hanusch Hospital, Vienna, Austria; b Orthopedic Hospital of Speising, Pediatric Department, Vienna, Austria; c Pediatric Orthopedic Institute n.a. H. Turner, Department of Foot and Ankle Surgery, Neuroorthopedics and Systemic Disorders, Parkovaya str., 64-68, Pushkin, Saint-Petersburg, Russia; d Department of Pediatric Orthopedic Surgery, Children Hospital, Tunis, Tunisia; e Institute of Medical Chemistry, Medical University of Vienna, Austria

**Keywords:** Fibrodysplasia ossificans progressiva, Congenital hallux valgus, Monophalangia, Progressive joint limitations, Imaging, *FBN1* gene mutation, *ACVR1* gene mutation

## Abstract

**Background:**

The clinical presentation, phenotypic characterization and natural history of fibrodysplasia ossificans progressiva (FOP) are diverse and the natural history of the disease is, to a certain extent, different from one patient to another.

**Methods:**

In a series of 11 patients (eight girls and three boys, aged 0 - 16 years), variable clinical presentations were the landmarks of these patients. At birth, all of our patients manifested short great toes in a valgus position. Marfan syndrome was the suggested diagnosis in three children aged 3 - 8 years and in two pre-adult patients. Clinical symptoms were torticollis, painful spine, and painful and marked limitation of the pelvic movements. Monophalangia associated with Marfanoid habitus was also a prevailing clinical presentation.

**Results:**

Our results were based upon the appearance of the earliest pathologic feature of FOP in correlation with the clinical presentation. In infants (0 - 1 year), three infants showed congenital hallux valgus and stiff spine. In the pediatric group (3 - 8 years), all children showed no mutation in the fibrillin-1 (*FBN1*) gene. Their prime presentation was a progressive torticollis with simultaneous development of erythematous subfascial nodules, most commonly located on the posterior neck and back. In pre-adult group (10 - 16 years), four patients presented with monophalangia associated with painful movements because of the progressive heterotopic ossification of the spine and the weight bearing zones and marked elevation of alkaline phosphatase. Genetic confirmation has been performed in six patients who manifested the classical mutation of the *ACVR1* gene. The rest of the patients were assessed via clinical and radiographic phenotypes.

**Conclusion:**

The early recognition of FOP can be performed by noticing the short halluces and thumbs at early infancy and later on the high alkaline phosphatase activity in areas of heterotopic ossification. Misconception of FOP is of common practice and eventually unnecessary diagnostic biopsies might deteriorate the progression of the condition. The detection of *ACVR1* gene mutation was a confirmatory procedure. Interestingly, the timing of the onset and the location of progressive heterotopic ossifications were extremely variable and confusing among our group of patients.

## Introduction

Fibrodysplasia ossificans progressiva (FOP) is a rare disorder of connective tissue differentiation that is characterized by congenital malformation of the great toes and progressive heterotopic ossification of tendons, ligaments, fascia and skeletal muscle [1, 2].

This disease is produced by a mutation in one of the copies of the gene that encodes the receptor I of the bone morphogenetic protein, called activin type 1 receptor or ACVR1 [3].

Usually, FOP begins in childhood as painful, erythematous subfascial nodules, most commonly located on the posterior neck and back. The individual nodules occasionally resolve, but more often progressively worsen and eventually mature into heterotopic bone. The diagnosis can be readily established while assessing the progressively developing subfascial nodules or ossification. Malformation of the great toes was reported in almost all newborns affected by FOP, meaning that congenital hallux valgus malformation is the earliest and most typical phenotypic characteristic [4].

Clinicians should be aware of the relationship between FOP and the great toe deformity as the earliest alarming sign.

Children with FOP seem ostensibly normal at birth except for malformation of the great toes (congenital hallux valgus). Sadly speaking, these children succumb to vicious and progressive episodes of heterotopic ossification that pathologically transform the normal soft connective tissues into bones. These pathological ossifications turned these children into a state of lifelong disability.

## Materials and Methods

The study protocol was approved by the Medical University of Vienna (Ethics Committee, EK Nr: 921/2009). Informed consents were obtained from the patients’ guardians. The original material comprised 11 patients (seven girls and four boys, aged 0 - 16 years) of different ethnic origins. They were clinically and radiologically evaluated at the Orthopedic Hospital of Speising, Department of “Bone disorders”, Vienna, Austria and within the collaboration research partnership with the Pediatric Orthopedic Institute n.a. H. Turner, Department of Foot and Ankle Surgery, Saint-Petersburg, Russia and the Pediatric Orthopedic Surgery Department, Children Hospital, Tunis, Tunisia.

We subdivided our patients into three groups (A, B and C) in accordance with age of diagnosis and the variable clinical phenotypes.

### Group A (infants aged 0 - 1 year)

Three infants aged 0 - 1 year (two males and one female) were brought to our department because of congenital hallux valgus. In this group, the parents’ awareness regarding the deformed big toes was the principal motive to seek advise. These families were assured by other physicians that such deformities are just a physiological variation. In addition, they manifested microdactyly of the big toes and the thumbs. The bilateral and the symmetrical involvement of the foot and thumb deformities was the baseline to arrange for skeletal survey.

### Group B (pediatric patients aged 3 - 8 years)

Three children presented with Marfanoid habitus, progressive torticollis with simultaneous development of erythematous subfascial nodules, most commonly located on the posterior neck and back. The diagnosis of Marfan syndrome was established and these children underwent genetic analysis to detect mutation in the fibrillin-1 (*FBN1*) gene. These children presented with torticollis associated with swellings over the neck and on the dorsal aspect of the trunk, shoulder girdle, and eventually in the proximal parts of the limbs. These events were observed in the group of patients less than 10 years of age. The swellings were initially fluctuating and painful, covered by erythematous and edematous skin, later converted into painless and wooden in consistency, involving the paravertebral regions especially cervical and the proximal limbs. These lesions extended progressively to other parts of the body. All patients manifested generalized muscular pain associated with osseous consistency.

### Group C (pre-adult group aged 10 - 16 years)

In two young adult patients, monophalangia associated with extreme restriction and mobility of the spine were the main presenting deformities. The bony abnormalities of the spine were mis-diagnosed initially as exostoses.

In a 14-year-old girl with Marfanoid habitus and ligamentous hyperlaxity, the diagnosis of Marfan syndrome was established. Marfan syndrome has been shown to be caused by mutations of the *FBN1* gene on chromosome 15. Though, no mutations have been encountered in this girl. Sadly speaking, short/hypoplastic halluces of the big toes were completely overlooked (malformed big toes with superimposed ankyloses of the proximal phalanx with the metatarsal of the first digit of the foot). At the age of 6 years, she developed subcutaneous lumps along her lumbar spine, which were provoked by trauma, and within a period of several months, there was a radiological evidence of small spots of ossification. Clinical examination showed a patient with Marfanoid habitus associated with hypoplastic hallux valgus (short toes in valgus position) associated with bilateral flexion deformities of the thumbs overwhelmed by a severe rigid spine with feasible and palpable bars of ossification. Revising her laboratory tests, alkaline phosphatase levels in blood were four times greater than normal in the period of 10 weeks after the inciting trauma.

A 10-year-old girl presented with progressive painful limitation of range of motion of the left hip following several weeks of repetitive trauma to her pelvis. She had no history of painful episodes or swellings, but discrete nodules over the trunk were evident. Because of her Marfanoid habitus and ligamentous hyperlaxity, the clinical diagnosis of Marfan syndrome was established, but there was no mutation in the *FBN1* gene. Clinical examination showed a patient with Marfanoid habitus associated with hypoplastic hallux valgus (short toes in valgus position) associated with bilateral flexion deformities of the thumbs. The overall clinical and radiographic phenotypes were in favor of FOP.

## Results

### Group A (infants aged 0 - 1 year)

**Figure 1:**
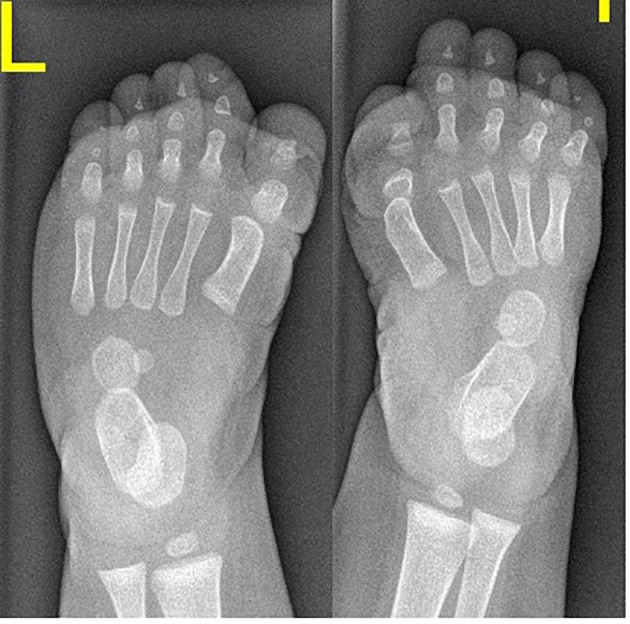
AP radiograph of a 6-month-old girl showed the characteristic shortening and broadening of the first metatarsal which was angulated medially with hypoplasia of the basal phalanx of the first toe - triangular distal phalanx. These features are the earliest in the diagnosis of FOP, making the diagnosis possible before the appearance of soft tissue calcification.

AP radiograph of a 6-month-old girl showed the characteristic shortening and broadening of the first metatarsal which was angulated medially with hypoplasia of the basal phalanx of the first toe - triangular distal phalanx. These features are the earliest in the diagnosis of FOP, making the diagnosis possible before the appearance of soft tissue calcification (Fig. 1, Table 1).

**Table 1:** Diversity of Clinical Presentations in Children and Adults With FOP

Patients	Age at which diagnosis was made, sex	Age at which soft tissue calcification first noted	Findings that led to diagnosis	Microdactyly of big toes and thumbs	Torticollis	Genetic testing results	Alkaline phosphatase levels	Surgical procedures	Other manifestations
Patient 1	6 months old, female	-	Congenital halux valgus	+	-	Negative for *ACVR1* gene mutation	Normal	None	1) Congenital flexion deformity of the thumbs 2) Unusual stiffness of the spine
Two patients of same age group	6 - 11 months old, two males	-	Congenital hallux valgus	+	-	Positive *ACVR1* mutation (R206H)	Normal	None	Congenital flexion deformity of the thumbs
Patient 4	5 years old, female	Three years	Congenital hallux valgus	+	++	Was tested primarily for *FBN1* gene which was negative. Then, positive for *ACVR1* gene mutation (R206H)	Two times greater than normal	None	Marfanoid habitus
Two patients same age group	7 - 8 years old, two males	Four years	Congenital hallux valgus	-	+++	Was tested primarily for *FBN1* gene and showed negative results	Two times greater than normal	None	Marfanoid habitus with extreme stiffness of the spine and pain
Patient 7	7 years old, female	Three years	Congenital hallux valgus	None	Torticollis associated with swellings over the neck	Positive *ACVR1* gene mutation (R206H)	Three times greater than normal	None	Marfanoid habitus associated with painful hips and knees
Patient 8	14 years old, female	Seven years	Monophalangia of the big toes	Monophalangia of the big toes	++	No genetic tests performed	Three times greater than normal	None	Painful ankylosis of all major joints
Patient 9	13 years old, female	Ten years	Monophalangia of the big toes	Monophalangia of the big toes	++	Positive *ACVR1* gene mutation (R206H)	Three times greater than normal	None	Extra-articular ankylosis of the left knee
Patient 10	14 years old, female	Seven years	Malformed big toes with superimposed ankyloses of the proximal phalanx with the metatarsal of the first digit of the foot	Severe microdactyly of the big toes	++	Was tested primarily for *FBN1* gene and showed negative	Alkaline phosphatase levels were four times greater than normal in the period of 10 weeks after the inciting trauma	None	Clinically diagnosed as Marfan syndrome
Patient 11	16 years old, female	Ten years	Congenital hallux valgus associated with Marfanoid habitus	++	-	Positive *ACVR1* gene mutation (R206H)	Alkaline phosphatase levels were four times greater than normal in the period of 12 weeks after the inciting trauma	None	Massive heterotopic ossification around the pelvis and the upper shaft of the left femur and the ischium pubis with eventual development of extra-articular ankyloses

**Figure 2:**
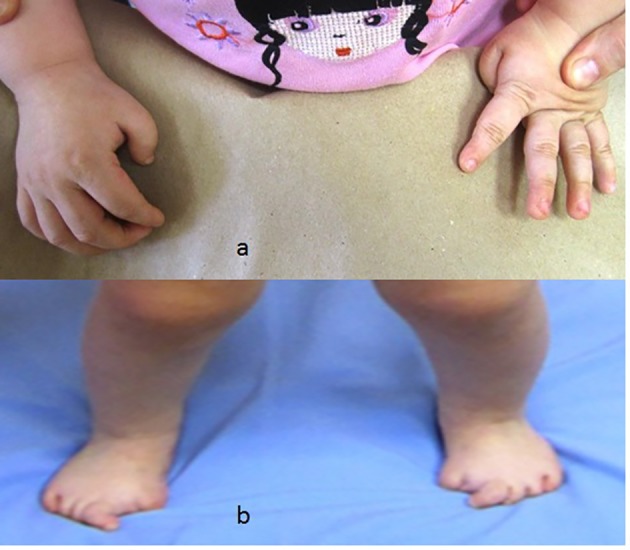
(a) Congenital flexion deformity of the thumbs is another clue for diagnosis. (b) Severe bilateral and symmetrical shortening of the big toes associated with flexion deformities of the second toes respectively.

Congenital flexion deformity of the thumbs is another clue for diagnosis (Fig. 2a) and severe bilateral and symmetrical shortening of the big toes associated with flexion deformities of the second toes respectively (Fig. 2b).

### Group B (pediatric patients aged 3 - 8 years)

**Figure 3:**
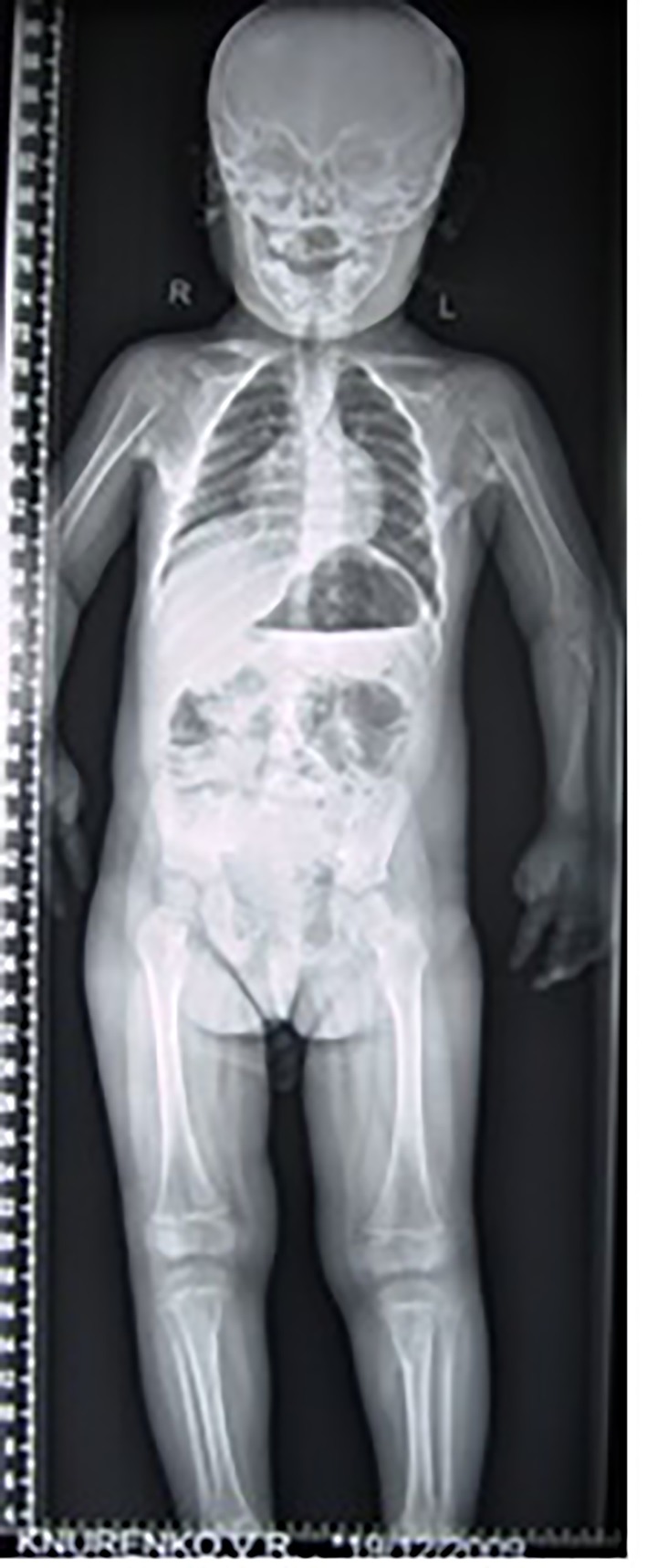
AP thorax radiograph showed the columns and plaques of ectopic bone in a 7-year-old girl with FOP and situs inversus (note the direction of the apex of the heart). Opacities in the muscles and near the tendons insertions were encountered in all patients with progression from proximal to distal and formation of a true bony bridge between various parts of the skeleton was evident (arrows).

Three children presented with Marfanoid habitus, progressive torticollis with simultaneous development of erythematous subfascial nodules, most commonly located on the posterior neck and back. The clinical diagnosis of Marfan syndrome was established and these children underwent genetic analysis to detect mutation in the *FBN1* gene which revealed negative in all cases. The hallux valgus and the short thumbs were not noted with the consequence of a delayed diagnosis. AP thorax radiograph showed the columns and plaques of ectopic bone in a 7-year-old girl with FOP and situs inversus (note the direction of the apex of the heart). Opacities in the muscles and near the tendons insertions were encountered in all patients with progression from proximal to distal and formation of a true bony bridge between various parts of the skeleton was evident (arrows) (Fig. 3).

### Group C (pre-adult patients aged 10 - 16 years)

**Figure 4:**
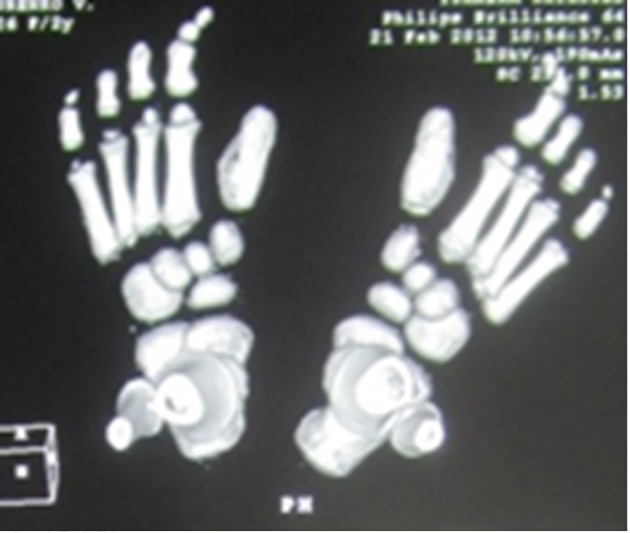
Reformatted CT scan in a 14-year-old girl showed monophalangia with subsequent development of severe shortening of the first metatarsals (secondary to abnormal fusion of the epiphyses).

Reformatted CT scan in a 14-year-old girl showed monophalangia with sebsequent development of severe shortening of the first metatarsals (secondary to abnormal fusion of the epiphyses) (Fig. 4). Progressive episodes of heterotopic ossifications typically lead to ankylosis of all major joints of the axial and appendicular skeleton, rendering movement impossible, associated with marked elevation in the blood alkaline phosphatase level.

**Figure 5:**
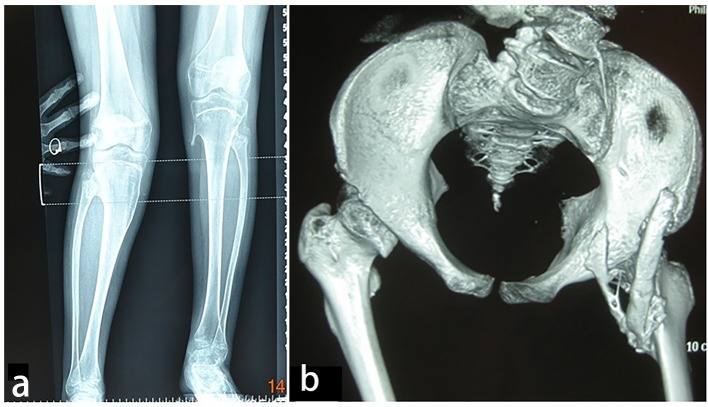
(a) Progressive episodes of heterotopic ossifications typically lead to ankylosis of all major joints of the axial and appendicular skeleton, rendering movement impossible. AP radiograph of the knees showed a 13-year-old girl presented with progressive painful limitation of range of motion of the both knees with progressive extra-severe articular ankylosis of the left ankle joint. (b) 3D reconstruction CT scan of the pelvis of the same patient (13-year-old) showed two ossified bands originated from the posterior aspect of the right iliac bone, run downwards and where both got fused and inserted beneath the femoral neck adding extra dilemma to the patient.

AP radiograph of the knees showed a 13-year-old girl presented with progressive painful limitation of range of motion of the both knees with progressive extra-severe articular ankylosis of the left ankle joint (Fig. 5a). 3D reconstruction CT scan of the pelvis of the same patient showed two ossified bands originated from the posterior aspect of the right iliac bone, run downwards and where both got fused and inserted beneath the femoral neck adding extra dilemma to the patient (Fig. 5b).

**Figure 6:**
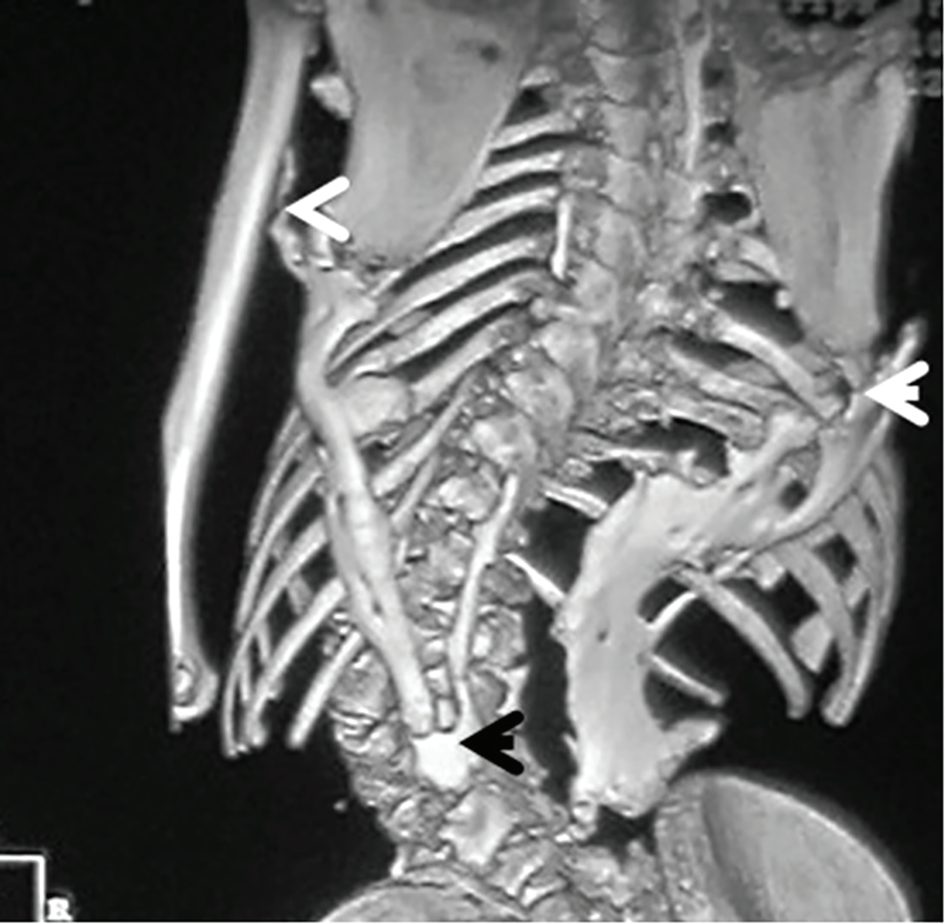
3D reconstruction CT scan in a 16-year-old girl showed diffuse ankylosing ossification of the inferior margins of the scapulae with that of the spine. On the right side, diffuse ankylosing ossification was noted which extended from the inferior margin of the right scapula with a downward manner to cover the posterior aspect of the ribs (7 - 12) until it got ankylosed with the right margin of S1. On the left side, a longitudinal bar of ossification fused superiorly with the inferior margin of the left scapula with downward extension to involve the posterior aspect of the ribs (6 - 12) and bifurcated to two divisions: one extended upward to involve the shaft of the left humerus (arrow head) and the other branch fused with another bar of ossifcation which run in parallel to other bar, which originated from the spinous process of T11 and extended downwards to fuse with the other ossifcation bar at the level of the spinous process of L4 with eventual formation of a dreadful ankylosis (black arrow) (Fig. 3). These progressive episodes of heterotopic ossifications typically lead to dreadful ankylosis of all major joints of the axial and appendicular skeleton, rendering movement impossible, associated with marked elevation in the alkaline phosphatase level (three times greater than normal).

3D reconstruction CT scan in this 16-year-old girl showed diffuse ankylosing ossification at the inferior margins of the scapulae with that of the spine. On the right side, the diffuse ankylosing ossification was noted to extend from the inferior margin of the right scapula with a downward manner to cover the posterior aspect of the ribs (7 - 12) until it got ankylosed with the right margin of S1. On the left side, a longitudinal bar of ossification fused superiorly with the inferior margin of the left scapula with downward extension to involve the posterior aspect of the ribs (6 - 12) and bifurcated to two divisions: one extended upward to involve the shaft of the left humerus (arrow head) and the other branch fused with another bar of ossification which run in parallel to other bar, which originated from the spinous process of T11 and extended downwards to fuse with the other ossifcation bar at the level of the spinous process of L4 with eventual formation of a dreadful ankylosis (black arrow) (Fig. 6). Revising her laboratory tests, alkaline phosphatase levels were four times greater than normal in the period of 10 weeks after the inciting trauma.

**Figure 7:**
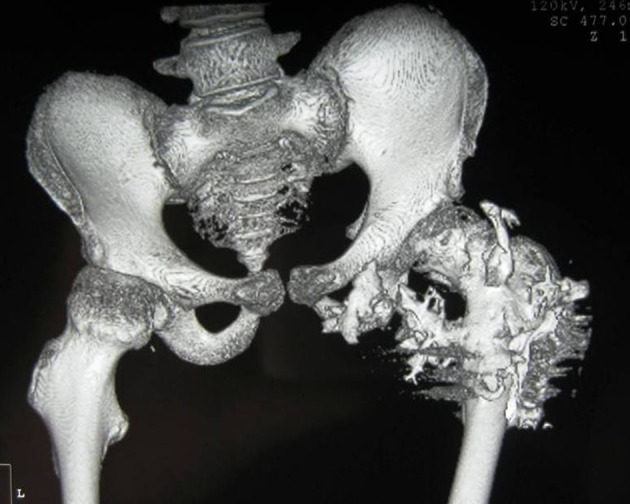
3D reconstruction CT scan of the pelvis in a 15-year-old female patient showed massive heterotopic ossification around the pelvis and the upper shaft of the left femur and the ischium pubis with eventual development of extra-articular ankyloses (firm bridging from the femur to the pelvis) associated with massive ossification of the soft tissue (i.e. heterotopic ossification formed in the soft tissues above and below the greater trochanter of the femur).

3D reconstruction CT scan of the pelvis in a 10-year-old girl showed massive heterotopic ossification around the pelvis and the upper shaft of the left femur and the ischium pubis with eventual development of extra-articular ankyloses (firm bridging from the femur to the pelvis) associated with massive ossification of the soft tissue (i.e. heterotopic ossification formed in the soft tissues above and below the greater trochanter of the femur) (Fig. 7).

To summarize our results, in the infants group (0 - 1 year), the parents’ awareness regarding the deformed big toes was the corner stone to seek advise. These families were assured by other physicians that such deformities are just a physiological variation.

In the pediatric group of patients (3 - 8 years), there was a delay in establishing the diagnosis because the congenital hallux valgus and the short thumbs were completely ignored by physicians. The marfanoid habitus was a confusing feature in this group and the diagnosis of Marfan was given to the parents. All were genetically tested for mutation in the *FBN1* gene which revealed negative.

In the pre-adult group (10 - 16 years), routine biochemical evaluations of bone mineral metabolism were normal. Though, bone remodeling rates and blood alkaline phosphatase activity were increased in four of our patients. The medical intervention was supportive and no surgical release for joint ankylosis was planned. Osteotomy of heterotopic bone to mobilize joints is uniformly counterproductive because excessive heterotopic ossifications develop at the site. Prophylactic measures to avoid episodic disease flare-ups which might be triggered via trauma, viruses as well as immunizations are always considered as a fundamental tool of disease flare-ups.

Not all patients underwent genetic testing because of logistical reasons, but seven patients were genetically tested. Six patients manifested the classical *ACVR1* gene mutation and one patient showed no mutation in the *ACVR1* gene. But in this one and in the rest of our patients, the diagnosis was confirmed through the clinical and the radiographic phenotypes (Table 1).

## Discussion

Rosenstirn collected 119 cases from the literature and added one of his own [5]. Most of the cases are sporadic, though an autosomal dominant pattern of inheritance has been documented in several reports. High levels of alkaline phosphatase are usually found in areas of ectopic ossification. The muscle fibers undergo secondary atrophic and degenerative changes. Later, calcification and ossification of the involved mesodermal tissues take place. On occasion, it may be difficult to distinguish FOP from osteogenic sarcoma.

FOP is characterized by progressive calcification and ossification of the muscles, soft tissues, tendons and aponeuroses and the fascia and connective tissues [6]. During the first weeks, painful erythema, swelling, warmth and tenderness are noted (early lesions). After several weeks, the swelling begins to subside (intermediate lesions). There is a decrease in pain, erythema and tenderness but an increase in induration. After approximately several weeks, the swelling disappears and there remains a hard, non-tender lesion that is visible radiographically as a new area of ossification (late lesions) [7]. Severe disability subsequently emerged in all our patients, since the course of the disease was of steady progression with periods of remissions with waves of acute exacerbations. Eventually all our patients become disabled. There is no specific therapy. Though, a trial of treatment with adrenocorticotrophic hormones had given dubious results in some patients. Beryllium has been tried and found to be of doubtful benefit. The progress of the disease can not be arrested by means of bisphosphonate. Operative excision has been disappointing as the surgical trauma has aggravated the condition, and more extensive bone has reformed [8]. Biopsies often trigger a clinical flare-up, but the mechanism is not understood.

The primary congenital skeletal abnormality in patients with FOP is malformation of the great toes. The toes are short, tend to be in a valgus position, and have an abnormally shaped proximal phalanx. They may become monophalangic if the abnormal epiphyses fuse. Often, no attention is given to the toe deformity until painful nodules or ossification develop. Some patients have clinically abnormal short thumbs due to short first metacarpals [9]. In most cases, there is an absent skin crease, a single phalanx and deviation of the toe laterally [2, 7].

The skeletal muscles are fundamentally normal, though the basic defect resides in the connective tissues. Thus the term myositis ossificans progressiva is a misnomer. In atypical case, swellings first appear in the neck or in the dorsal aspect of the trunk, in the shoulder girdle and eventually in the proximal parts of the limbs. The site of involvement may also be determined by local injury. These swellings are usually small, although at times, they may be as large as an egg or an apple. In the early acute phase, they are painful, locally tender, and slightly warm and associated with a low-grade fever. Swellings may be cyst-like and fluctuating or they may be firm from onset. Often they are attached to the deep fascia and the overlying skin is normal and loose, but on occasion, they may be ill-defined and not adherent to the deep fascia [10]. FOP as an osteomyelitis-like syndrome has been described [11].

Torticollis is a common presenting complaint, the head is tilted to one side and there are painful swellings in the region of the sternocleidomastoid muscle. Flexion of the neck is limited when the ligamentum nuchae is involved. Motion of the temporo-mandibular joint is diminished, with affection of the masseters [12].

Connor et al reported a family where individuals in three generations were affected [13]. They reviewed the evidence for autosomal dominant inheritance which includes concordant monozygotic twins, a paternal age effect for presumed new mutations, and several instances of parent to child transmission, including male to male transmission. Shore et al identified linkage of FOP to chromosome 2q23-q24, a genetic interval including the *ACVR1* gene [14]. Sequence analysis of all ACVR1 protein-coding exons and splice junctions identified a heterozygous mutation (R206H; 102576.0001) in all examined familial individuals, including all five families used for linkage analysis and in 32 of 32 sporadic FOP patients with unambiguous clinical features.

Kaplan et al [15] suggested, from homology with the decapentaplegic (dpp) locus of Drosophila, which is caused by an abnormality of a gene in the TGF-beta family, that bone morphogenetic proteins 2A and 2B may be involved in human FOP.

Bruni et al [16] reviewed the use of ethane-1-hydroxy-1,1-diphosphonate (EHDP) in this condition. Bar Oz and Boneh [17] reported a case treated for 10 years on this regime. There was gradual progression of the disease but no typical bouts of swelling, reddening or hardening of areas over skeletal muscle, which is usually seen in untreated cases.

### Conclusion

In our practice, we observed that a large number of patients were treated in accordance with the presenting deformity. The associated unnoticeable abnormalities such as congenital hallux valgus/flexion deformity of the thumbs were totally ignored. Thus, incorrect diagnoses were the reason behind the progressive and serious exacerbation of the lesions, leading frequently to permanent disability. FOP has been considered as an embarrassing puzzle to inexperienced physicians. Other major syndromic entities which might mimic the phenotype of FOP are 1) some patients exhibit the Marfanoid habitus; 2) progressive osseous heteroplasia (in these patients the great toe is normal); 3) multiple cartilaginous exostoses (not associated with subcutaneous ossifications); and 4) pseudo-pseudohypoparathyroidism.

Physicians who are experienced in approaching definite diagnosis relying on clinical and radiographic phenotypes could help in reducing the dreadful course of the disease through proper monitoring despite the absence of definitive treatment. The most likely explanation for the high rates of misdiagnoses is that the phenotypic characterizations of the disease are diverse and confusing. In addition, the lack of awareness and restricted knowledge in orthopedic deformities in connection with syndromic entities plays the major role in misconception/management of such deformities.
